# Aberrant LncRNA Expression Profile in a Contusion Spinal Cord Injury Mouse Model

**DOI:** 10.1155/2016/9249401

**Published:** 2016-09-04

**Authors:** Ya Ding, Zhiwen Song, Jinbo Liu

**Affiliations:** ^1^Department of Orthopedics, The Third Affiliated Hospital, Soochow University, Changzhou, Jiangsu, China; ^2^Department of Orthopedic Surgery, Fuyang People's Hospital, Anhui Medical University, No. 63 Luci Street, Fuyang City, Anhui 236004, China

## Abstract

Long noncoding RNAs (LncRNAs) play a crucial role in cell growth, development, and various diseases related to the central nervous system. However, LncRNA differential expression profiles in spinal cord injury are yet to be reported. In this study, we profiled the expression pattern of LncRNAs using a microarray method in a contusion spinal cord injury (SCI) mouse model. Compared with a spinal cord without injury, few changes in LncRNA expression levels were noted 1 day after injury. The differential changes in LncRNA expression peaked 1 week after SCI and subsequently declined until 3 weeks after injury. Quantitative real-time polymerase chain reaction (qRT-PCR) was used to validate the reliability of the microarray, demonstrating that the results were reliable. Gene ontology (GO) analysis indicated that differentially expressed mRNAs were involved in transport, cell adhesion, ion transport, and metabolic processes, among others. Kyoto Encyclopedia of Genes and Genomes (KEGG) enrichment analysis showed that the neuroactive ligand-receptor interaction, the PI3K-Akt signaling pathway, and focal adhesions were potentially implicated in SCI pathology. We constructed a dynamic LncRNA-mRNA network containing 264 LncRNAs and 949 mRNAs to elucidate the interactions between the LncRNAs and mRNAs. Overall, the results from this study indicate for the first time that LncRNAs are differentially expressed in a contusion SCI mouse model.

## 1. Introduction

Spinal cord injury (SCI) has become a global burden, influencing the quality of life and triggering serious socioeconomic consequences. Having SCI means being confined to a wheelchair and a lifetime of medical disease [1]. Repairing SCI is challenging due to multiple factors, including extensive cell loss, axonal disruption, growth-inhibiting molecules in the scar, and a lack of growth-promoting molecules [2]. Several studies have indicated that changes in various cellular events are significantly associated with SCI. Among these changes is the dysregulated gene expression of specific molecules. Large-scale gene expression studies had revealed that numerous protein-coding genes are differentially expressed in SCI, a portion of which was demonstrated to play pivotal roles in SCI [3, 4]. However, abnormal gene expression is a highly complex process.

Recently, increasing evidence has indicated that noncoding RNAs possess significant regulatory functions in SCI [5–9]. Long noncoding RNAs (LncRNAs) have various functions [9] and range from 200 bp up to several kilobases in length; this length was determined from a convenient practical cut-off in RNA purification protocols which excludes other RNAs [5]. Several studies have revealed that LncRNAs are especially enriched in the central nervous system and are implicated in several neurological diseases [10–12].

To date, no studies have focused on the differential expression of LncRNAs in an SCI model. In this study, we surveyed the temporal expression of LncRNAs in the spinal cord following contusive SCI in* ICR* mice by microarray analysis and validated the results with quantitative real-time polymerase chain reaction (qRT-PCR). Our research provides the first evidence of an aberrant LncRNA expression profile in SCI.

## 2. Materials and Methods

### 2.1. Ethics Statement

All experimental procedures were conducted in conformity with institutional guidelines for the care and use of laboratory animals, and protocols were approved by the Institutional Animal Care and Use Committee in Soochow University, Jiangsu, China. This institution approved this study (Permit number: SYXK2012-0045). Although the license was Chinese version, we upload the figure of license in the attached file (S1 license_pdf). Mice were provided with food, sterile water, and the appropriate ambient temperature in all experiment procedures and executed using the broken neck method under the deeply anesthetized condition. After death, the mice were incinerated and buried in the assigned place. A small funeral was held in the end.

### 2.2. Animals

Male* ICR* mice (20–25 g) aged 6–8 weeks were used in these experiments (Carvens Laboratory Animal Technology Co., Ltd., Changzhou, China). All of the mice were allowed preoperative environmental adaptation for 1 week with normal circadian rhythms (one 12-hour light-dark cycle) and had free access to water and food. The mice were bred in cages (3 mice/cage).

### 2.3. Spinal Cord Injury Surgery

Before the surgery, the skin preparation by scissor and disinfection of the operative region by povidone iodine were performed. The mice were deeply anesthetized with intraperitoneal pentobarbital sodium (50 mg/kg body weight). According to the anatomy of the mice, T10 vertebrae were the highest of the back when the mouse was in prone position and then we labeled T8 and T12 vertebrae proximally and distally. Subsequently, an incision was made on the mid-line of the back over the spinous processes from T8 to T12 vertebrae. Then, we exposed and separated the paravertebral muscles from the vertebra. Following a laminectomy at the 10th thoracic spinal vertebrae (T10), the underlying spinal cord at T10 was exposed, and the spinal cord was contused with a Multicenter Animal Spinal Cord Injury Study (MASCIS) Impactor weight-drop device, which uses a 5 g weight impact rod dropped from a height of 25 mm to produce a reliable contused SCI model. Following the injury, the muscle and skin were closed with absorbable sutures. According to previous studies, the mice were divided into five groups (three mice/group, sham operation, 1 day after injury, 3 days after injury, 1 week after injury, and 3 weeks after injury) and provided with food, sterile water, and the appropriate ambient temperature after the surgery. Meanwhile, tramadol hydrochloride (50 mg/kg body weight) was intraperitoneal for the postoperative analgesia. The mice were given manual bladder evacuations twice per day. The spinal cord located 5 mm proximally and distally to the injury epicenter was removed for RNA extraction.

### 2.4. Total RNA Extraction and Quality Control Assay

Total RNA was isolated using TRIzol reagent (Invitrogen, Canada) and purified using an RNeasy Mini Kit (Qiagen, Germany), which includes a DNase digestion treatment. RNA concentrations were determined based on the absorbance at 260 nm and quality control standards at *A*260/*A*280 = 1.8–2.1 using a NanoDrop 2000 (Thermo, US). RNAs from mice in the same groups were mixed to obtain equal masses for the microarray preparation.

### 2.5. Microarray Analysis

The Affymetrix GeneChip Mouse Exon 1.0 ST Array (Custom CDF), which contains 42,000 LncRNAs and 16,416 protein-coding transcripts, was employed in this study according to the manufacturer's protocols from the GeneChip platform by Affymetrix (Santa Clara, CA. US). The LncRNAs were collected from several well-known data sources, including RefSeq, Ensembl, UCSC, NOCODE, and the related data literature.

Affymetrix Expression Console (versions 1.3.1) was employed for data parsing, quality control, and LncRNA standardization according to the random variance model (RVM). The normalized signal intensities for the LncRNAs and mRNAs were filed as a log_2_ ratio.

### 2.6. Quantitative Real-Time PCR Assay

To further confirm the reliability of the microarray assay, qRT-PCR assays were performed. Briefly, single-stranded cDNA was synthesized using the RevertAid kit (Fermentas Life Science, Burlington, ON, Canada), according to the manufacturer's protocols, with random primers and 1 *μ*g RNA from the same samples used in the microarray. Real-time PCR was conducted using the SYBR Green q-PCR SuperMix (Bio-Rad, USA). The primers used are listed in Table 1. Each qRT-PCR reaction included 10 *μ*L SYBR Green q-PCR SuperMix, 0.5 *μ*L forward primer (10 *μ*M), 0.5 *μ*L reverse primer (10 *μ*M), and 1 *μ*L cDNA. The total volume was adjusted to 20 *μ*L with ddH_2_O. The following thermocycler parameters were used to generate the dissociation curve: (1) 95°C for 5 min; (2) 40 cycles of 95°C for 15 s, 60°C for 40 s, and 72°C for 20 s; and (3) 65 to 95°C. The 7500 System SDS software (ABI, USA) was used for acquiring the Ct values with manual thresholds. PCR amplifications were performed in triplicate for each sample. Gene expression levels were normalized relative to the expression of GAPDH using the ΔΔCT method. The gene expression levels between the groups were compared using 2^−ΔΔCT^ and Student's *t*-test. *p* values < 0.05 were considered statistically significant.

### 2.7. Series Test of Cluster Analysis

To profile the gene expression time series and ascertain the most probable set of clusters generating the observed time series, the series test of cluster (STC) algorithm was employed. The primary advantages of this method include the ability to take the dynamic nature of gene expression time series into consideration during clustering with a reliable method for identifying the number of distinct clusters [13, 14]. Briefly, the null hypothesis was defined such that the value of any past or future time point was independent. According to the null hypothesis, the number of genes in each trend conformed to a binomial distribution. Next, the number of predicted genes originally belonging to various trends was calculated by the means of replacement, with the various binomial distribution parameters under the null hypothesis trend being satisfied. Finally, the trend level significance was determined by calculating the probability that a variable obeyed the binomial distribution and was greater than or equal to the actual number of genes trends. The figures for the significant and nonsignificant trends were generated based on these results.

### 2.8. GO and KEGG Enrichment Analysis

GO analysis was utilized to identify the main processes and functional categories involved in the differentially expressed enrichment as previously described [15–18]. This method analyzed the number of genes present in a category on the microarray. Specifically, two-sided Fisher's exact test and *χ* ^2^ test were used to classify the GO categories and multiple comparison testing was performed by computing the false discovery rate (FDR) to correct *p* value (*p* < 0.0001). KEGG enrichment analysis was performed as described previously [19, 20] to investigate whether differentially expressed genes share similar biological functions. Two-sided Fisher's exact test and *χ* ^2^ test were selected to identify the significant pathways, and the threshold of significance was defined using the FDR and *p* value (*p* < 0.0001).

### 2.9. Dynamic LncRNA-mRNA Network

A dynamic LncRNA-mRNA network was constructed based on the correlation analysis between the differentially expressed LncRNAs and mRNAs as described previously [15, 21–23]. In the network analysis, the degree was defined as the sum of the links that one node has to all of the other nodes. The K-core in the graph theory was defined as a method of simplifying the analysis of graph topologies. The clustering coefficient was defined as a measure of the relationship between a gene and its neighboring genes. We calculated Pearson's correlation coefficient for each pair of genes and selected significant correlation pairs to build the network.

## 3. Results

### 3.1. Microarray Data

**Figure 1:**
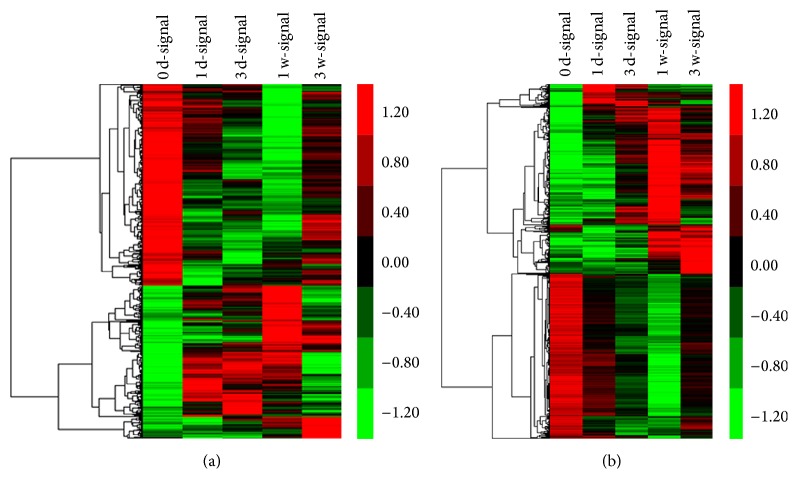
Heat maps and hierarchical clustering showed a remarkable differential expression of LncRNAs (a) and mRNAs (b) at five continuous time points after SCI. Red and green represented the high and low expression levels, respectively. Black indicated the mean expression levels. Each column represented a single group, and each row represented an LncRNA.

The genes with an FDR ≤ 0.05 and a fold change ≥ 2 were selected. The microarray data (Table 2) indicated that the LncRNA and mRNA expression dynamically changed from the initial injury to 1 day, 3 days, 1 week, and 3 weeks after injury. We discovered that the number of differentially expressed LncRNAs and mRNAs at 1 day after injury was similar to day 0. However, the expression strongly changed at 3 days and 1 week and appeared to decline at 3 weeks after injury. We integrated the differential LncRNA and mRNA expression at five time points and created a union set (Tables S1 and S2 in Supplementary Material available online at http://dx.doi.org/10.1155/2016/9249401). With this set, the heat maps and hierarchical clustering visually and dynamically demonstrated the differential expression of the LncRNAs and mRNAs (Figure 1). The microarray data have been approved and assigned in the National Center for Biotechnology Information Gene Expression Omnibus (GEO) [GSE67515: GEO].

### 3.2. qRT-PCR Validation Array

**Figure 2:**
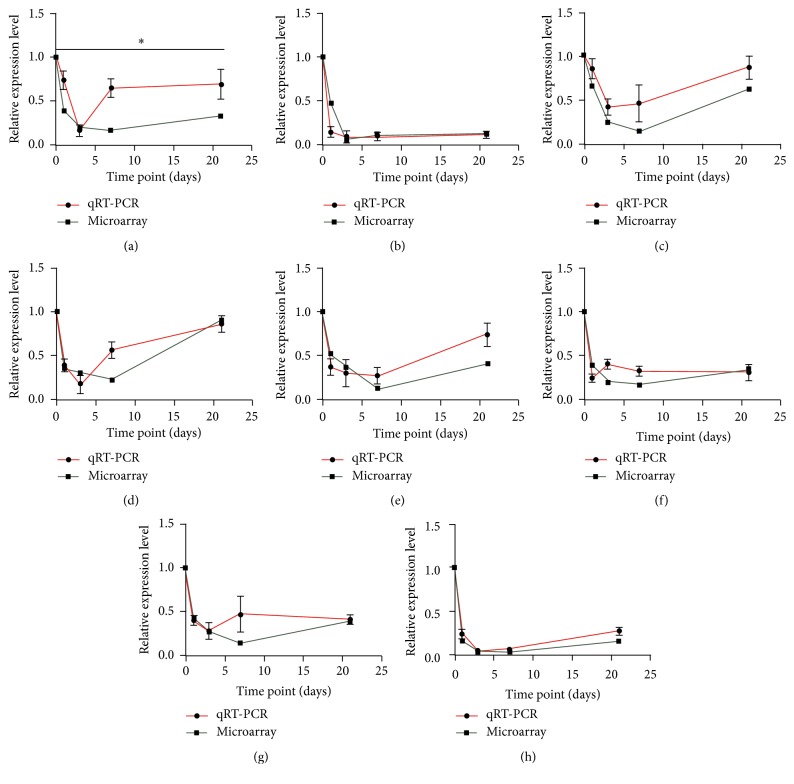
The validation of the reliability of microarray data via quantitative real-time PCR. Red curve represents the result of qRT-PCR, while the green curve represents the data of the LncRNAs microarray. *y*-axis indicates the relative expression level of LncRNAs which was normalized relative to GAPDH expression level by ΔΔCT method compared to the negative group (0 days) via 2^−ΔΔCT^ method. *x*-axis represents the days after spinal cord injury. (a) The horizontal line means that there is a disagreement between the microarray group and the quantitative real-time PCR group. (a–h) NONMMUT052085, NONMMUT038925, NONMMUT067118, NONMMUT051225, NONMMUT005924, NONMMUT070015, NONMMUT021928, and NONMMUT061607. *∗* represents *p* ≤ 0.05.

To further validate the reliability of the microarray data, qRT-PCR was performed. We used the NONCODE database (http://www.noncode.org/) to select eight LncRNAs that are highly expressed in the central nervous system (NONMMUT052085, NONMMUT038925, NONMMUT067118, NONMMUT051225, NONMMUT005924, NONMMUT070015, NONMMUT021928, and NONMMUT061607). As shown in Figure 2, the variation from the RT-PCR was similar to that of the microarray. Student's *t*-test indicates an agreement between the microarray data and qRT-PCR results with the exception of LncRNA NONMMUT052085. This finding indicates that our microarray data were reliable and can be used for bioinformatics analysis in the subsequent steps.

### 3.3. Series Test of Cluster Analysis

**Figure 3:**
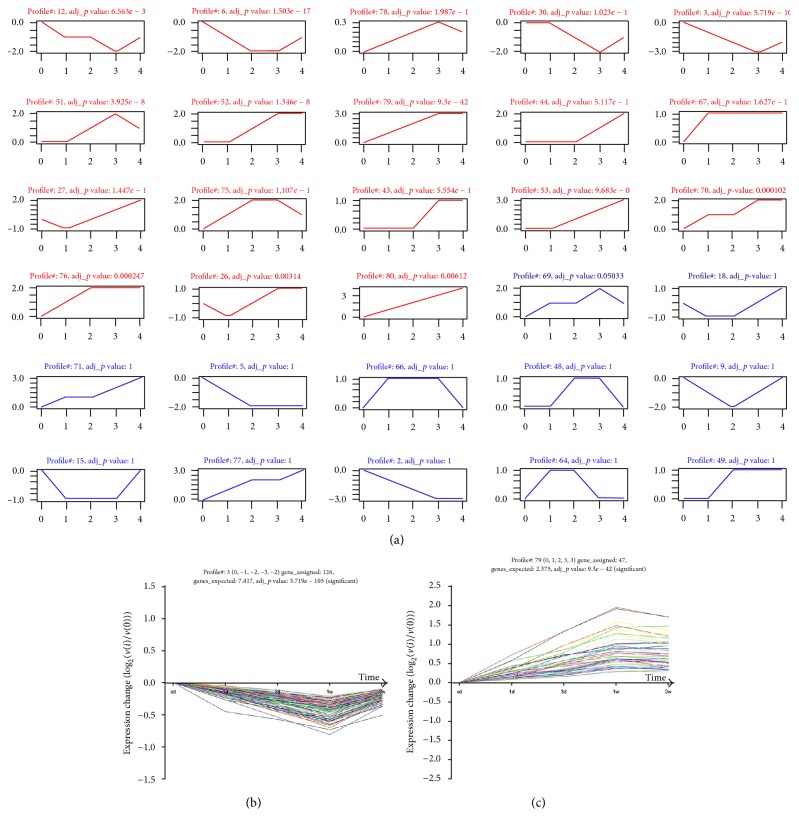
STC analysis for differential expression LncRNAs related to SCI. (a) The expression patterns of these differential expression LncRNAs were analyzed and eighty model profiles were employed for epitomization. Each coordinate axis represents an expression pattern. Each expression pattern corresponds to the trend of different LncRNAs transient expression. The red line in coordinate axis has statistical significance (adj_*p* value < 0.05) and the blue line does not. The upper number in the coordinate axis is the number of expression profiles and *p* value. The horizontal axis stands for the point in time after injury, and the vertical axis represents the LncRNAs expression level. The number of vertical axes does not represent the actual value of LncRNAs expression but the marker of gene expression level. (b and c) Two typical significant profiles were chosen to exhibit the specific pattern of gene expression. The curve showed individual gene expression profiles. The upper number in the coordinate axis first is the number of profile; genes_assigned represents the number of actual differential expression genes in this trend; genes_expected stands for the theoretical number of LncRNAs in this trend according to the random distribution; adj_*p* value represents the significant level of the ratio of the actual LncRNAs number to the theoretical number of genes in this trend. We defined the significance at adj_*p* value < 0.05.

**Figure 4:**
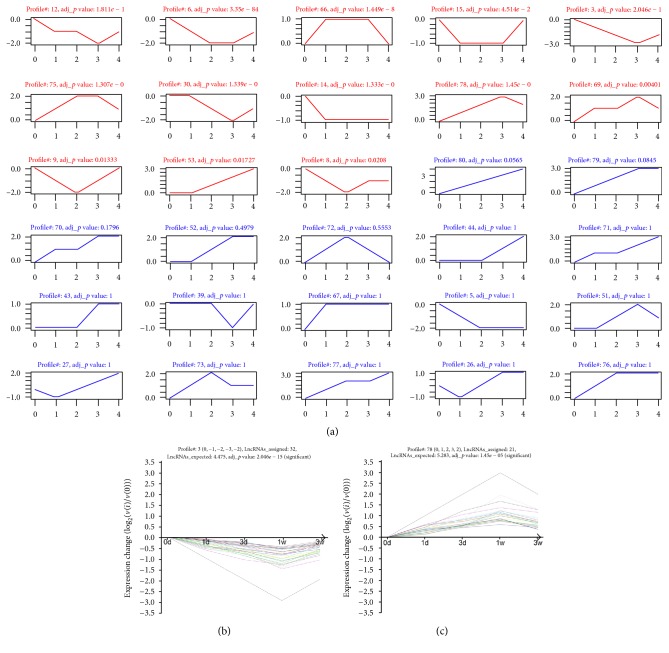
STC analysis for differential expression mRNAs related to SCI. (a) as is similar to Figure 3; it represents the expression patterns of these differential expression mRNAs. The coordinate axis and upper number were the same as Figure 3(a). (b and c) Two typical significant profiles were chosen to show the specific pattern of mRNAs expression. As an analogy to Figures 3(b) and 3(c), the coordinate axis and upper number were the same.

To further narrow the differential expression of the LncRNAs and mRNAs and seek the trend levels of significance among the differential expression profiles, STC was used to detect temporal expression patterns of significantly differentially expressed genes and to identify the cluster of diverse genes that have similar expression patterns after SCI (Tables S3 and S4). As illustrated in Figures 3 and 4, the differential expression profile consisted of eighty differentially expressed LncRNA clusters and eighty differentially expressed mRNA clusters. Each cluster represented a model trend profile and contained genes with similar expression trends. Among these clusters, eighteen mRNA clusters and thirteen LncRNA clusters exhibited significant expression trends. These significant trends exhibited a variety of model trends, including stabilization, gradual augment, and decline.

### 3.4. Functional Analysis of the Differentially Expressed mRNAs

**Figure 5:**
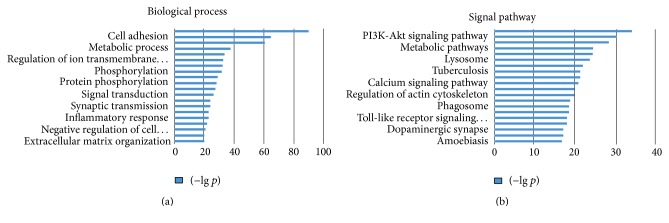
GO and KEGG enrichment analysis related to genes after SCI. (a) GO enrichment analysis was used to analyze genes having significant expression trend. Two-side Fisher's exact test and *χ* ^2^ test were employed and got *p* value. The vertical axis represents biological process in which mRNAs having significant expression trend related to SCI are involved. The green bar presented *p* value as −lg transformed pattern. (b) KEGG enrichment analysis was utilized to seek for the relative signal pathway in which mRNAs had significant expression trends related to SCI. Parallel to (a), the vertical axis represents signal pathway, and the green bar stands for *p* value as −lg transformed pattern.

GO and KEGG analyses were used to further evaluate the function of the genes that have significant expression trends. As shown in Figure 5, GO enrichment analysis, which identifies biological processes that the enriched transcripts are involved in, indicated that transport, cell adhesion, ion transport, metabolic process, innate immune response, and other significant biological processes were involved in SCI (Table S5). Additionally, KEGG enrichment analysis, which was performed to verify the significant module functions, revealed that neuroactive ligand-receptor interactions, the PI3K-Akt signaling pathway, focal adhesions, metabolic pathways, osteoclast differentiation, lysosomes, and other significant signal pathways were related to SCI (Table S6).

### 3.5. Dynamic LncRNA-mRNA Network

To discover the significant molecular mechanisms of the LncRNAs associated with the pathology of SCI, a dynamic LncRNA-mRNA network that contained 264 LncRNAs and 949 mRNAs was constructed (Figure S1 and Table S7). Interaction between these genes is noted when the quantification of LncRNA and mRNA interaction coexpression is greater than or equal to 0.997. In these maps, we identified various key LncRNAs with high degrees and K-cores, such as NONMMUT038518, ENSMUST00000145363, NONMMUT001318, NONMMUT035870, and NONMMUT054688, which might play important roles in SCI pathology. In this map, the size of the circle represents the ability of the gene to interact, according to the quantification of the degree.

## 4. Discussion

This study was the first to report the LncRNA expression profile in a mouse contusion SCI model. SCI was a complex biological process that involves many molecules and cell events. Although LncRNAs were once deemed the “noise” of the genome, LncRNAs have been shown to be enriched in the central nervous system and exerted a significant effect on its growth and development [24]. Hence, understanding the LncRNA expression profile was crucial for exploring potential LncRNA functions in SCI pathology.

As described in previous studies [25–27], the mechanical forces imparted on the spinal cord cause primary damage to the neural tissue, but a complex cascade of pathophysiologic processes imperiling adjacent, initially spared tissue rapidly caused secondary damage following the initial event. Such studies [28, 29] have identified a number of interrelated processes that are thought to contribute to the secondary damage after spinal cord primary injury, including alterations in microvascular perfusion, free radical generation and lipid peroxidation, necrotic and apoptotic cell death, and ionic homeostasis dysregulation. Among these changes, the most important alteration was necrotic and apoptotic cell death. Several studies [29, 30] have suggested that cell fate peaks at 1 day and 7 days after injury. Based on this observation, we added two time points at 3 days and 21 days after injury to identify the dynamical gene expression changes that occur. In this current study, the differential peaked at 7 days after injury. This finding might offer a clue for future research.

Animal models continued to play critical roles in the development of experimental research for SCI [31]. Injury reproducibility was an important characteristic of experimental SCI models because it limited the variability in gene expression outcomes. In this study, the contusion spinal cord injury model was classic and could mimic a clinical situation by using a MASCIS Impactor weight-drop device that employs a compression that causes bony fragments or extruded disk materials [32]. We produced a steady and reliable animal model, pooled RNA from injured spinal cords from 3 animals in each group to obtain total cellular RNA, and mixed the total cellular RNA at equal masses for the GeneChip preparations. However, only one GeneChip was subsequently used for each group, and we expanded the sample size (3 samples in each group) to guarantee reliable and precise microarray results, which correlated with the qRT-PCR results. To increase the reliability of the microarray results, the GEO database was used to identify results similar to those of our study. GSE45006 supports the reliability of our microarray results by blasting the mRNAs expression levels. We chose the significant profile 12 by series test of cluster analysis to blast to GSE45006 data and found they had 74.8% of the same change tendency of mRNAs.

After determining that the microarray results were reliable, RNAs that had significant changes in expression level (fold change ≥ 2) were chosen for the bioinformatics analysis. Cluster analysis of gene expression dynamics could offer linearity change trends for increasing and decreasing gene expression for future studies. This method represents gene expression dynamics as autoregressive equations, uses an agglomerative procedure to search for the most probable set of clusters given the available data, and considers the dynamic nature of gene expression time series during clustering with a reliable method for identifying the number of distinct clusters. Through this STC approach, one can extract gene sets with increasing and decreasing expression for specific studies.

For significantly dysregulated mRNAs, we examined potential LncRNA functions using GO enrichment and pathway analysis. GO analysis revealed that many of the genes with changed expression profiles were involved in transport, cell adhesion, ion transport, metabolic process, and innate immune response. Similarly, KEGG enrichment analysis demonstrated that the changed genes were involved in neuroactive ligand-receptor interactions, the PI3K-Akt signaling pathway, focal adhesion, and metabolic pathways. Then, subsequently, the dynamic LncRNA-mRNA network was constructed. This network was helpful for understanding the possible interactions and relationships between the differentially expressed LncRNAs and mRNAs.

The PI3K-Akt signaling pathway was classical pathway which was involved in apoptosis, protein synthesis, metabolism, and cell cycle and evidences indicated that this activated pathway could help in nerve regeneration [33, 34]. The mRNAs which associated with this pathway and have been contained in the dynamic LncRNA-mRNA network were extracted. For example, FGF1 was a neurotrophic factor which had high expression level in gliocyte cell and a powerful neuroprotective and neuroregenerative factor of the nervous system [34, 35]. In our study, this mRNA expression level was declined and had a dynamic relation with two LncRNAs (ENSMUST00000138093 and ENSMUST00000129688) in the network. Both had high expression level and declined in the SCI pathology. In humans, FGF1 was regulated by HOTAIR via upregulating miR-326 expression, forming the HOTAIR-miR-326-FGF1 axis [35]. This gave us inspiration for the future work.

However, there were many other signal pathways which could infect cell motility, cell proliferation, cell differentiation, regulation of gene expression, and cell survival. One of them was focal adhesion signal pathway. With the same method, the mRNAs were extracted, containing THBS1, SPP1, CAV1, MAPK10, and so on. THBS1 was involved in neuronal migration and adhesion, neurite outgrowth, and synaptogenesis, which draws our attention. THBS1 could decrease neuronal excitability via reducing AMPA receptors (AMPARs) and increasing glycine receptors (GlyRs) in synapses [36]. Some researches suggested that THBS1 helped the recovery of normal synaptic activity after injury responses [37] and even was a potential therapeutic target in the pathogenesis of Alzheimer's disease [38]. Many studies [39, 40] indicated that LncRNAs played key role in the neuronal degeneration disease which was involved in the progressive loss of structure or function of neurons, including death of neurons. In our study, THBS1 gradually increased and had contrary variation trend with the LncRNA NONMMUT027272 in the dynamic LncRNA-mRNA network. This could offer the idea in the future work.

However, some limitations in the current study should be noted. For example, the small numbers of samples in the microarray limit its reliability. We decreased the individual differences by mixing RNAs from different samples in the same group and increased the number of qRT-PCR validation genes. Additionally, given our research methods, we merely predicted the function of differentially expressed LncRNAs and were unable to determine exactly how these LncRNAs regulate target gene expression. LncRNA function can be validated by overexpression and RNA interference approaches. The molecular mechanisms can be investigated by RNA immunoprecipitation and chromatin immunoprecipitation, among others. In the future, we aim to further investigate and focus on LncRNA molecular mechanisms in SCI repair.

## 5. Conclusions

Overall, we demonstrated the differential expression profile of LncRNAs after SCI. To the best of our knowledge, our results will be helpful for understanding SCI pathology.

## Supplementary Material

S1 license pdf: The License Awarded by Soochow University.
Table S1: Differential Expression of LncRNAs and Annotation.
Table S2: Differential Expression of mRNAs and Annotation.
Table S3: mRNAs STC Increase or Decrease.
Table S4: LncRNAs STC Increase or Decrease.
Table S5: The Significant GO Terms for Differentially Expressed.
Table S6: The Significant Pathways of Differentially Expressed Genes.
Table S7: The Quantity of the mRNA and LncRNA Network.
S1 Fig: The dynamic network between LncRNAs and mRNAs. In this map, the circles with a green line represented LncRNAs, and the other circles represented mRNA. The lines between circles indicated regulation between these genes. The size of the circles indicated the ability of interaction between the genes. This ability was quantified by the degree, which was defined as the connective number between genes. The color was in accordance with the results of the clustering analysis, which was defined as the K-core. The same K-core indicated the similarity and correlation of function between genes. The red color representing the maximal K-core indicated the strongest regulation level in this network.

## Figures and Tables

**Table 1:** The list of primers for qRT-PCR.

Primer	Sequence (5′ to 3′)	Base
mmu-NONMMUT052085 (forward)	GGTGGTTTAGTCATGCACGC	20
mmu-NONMMUT052085 (reverse)	TGCAGAGGACTATGGAGGCA	20
mmu-NONMMUT038925 (forward)	TCTCCTGTTCCCACAAGACC	20
mmu-NONMMUT038925 (reverse)	GTAGAATGATGTGCGTGCCTG	21
mmu-NONMMUT067118 (forward)	AGGCTTTCATTTCTCGCCACT	21
mmu-NONMMUT067118 (reverse)	CACTCTTGGTGACGAGGAACAC	22
mmu-NONMMUT051225 (forward)	TTCCCGCACACCCAAGTTTA	20
mmu-NONMMUT051225 (reverse)	TGTGCACCCAAAGCCTGTAA	20
mmu-NONMMUT005924 (forward)	GGGTGGTTCGTGATGAGTGT	20
mmu-NONMMUT005924 (reverse)	TGCAGAACAGAGCCCTTAGC	20
mmu-NONMMUT070015 (forward)	AAGGAGGGGAACAACAACCC	20
mmu-NONMMUT070015 (reverse)	CACCAGCTTAGCTCCTCCAC	20
mmu-NONMMUT021928 (forward)	TGTGAGGATGCCTTCTGCTC	20
mmu-NONMMUT021928 (reverse)	TAAGTGGGCAAAGCGGAGAT	26
mmu-NONMMUT061607 (forward)	TCTCCATTATACATGCTGATGCCT	24
mmu-NONMMUT061607 (reverse)	GGTCGAAATATTTTAGATGGAAGCA	25

**Table 2:** The differential expression profile of LncRNAs and mRNAs.

Group	LncRNA		mRNA	
	Upregulation	Downregulation	Upregulation	Downregulation
1 day after injury	164	181	309	149
3 days after injury	212	290	541	532
7 days after injury	326	565	1045	1132
21 days after injury	141	40	746	104

Group of experiment was according to the pathology of SCI. The number of this table represented the quantity of differential expression of LncRNAs and mRNAs by using without injured spinal cord as the control.
