# Supplementary figures and images for: Aberrant LncRNA Expression Profile in a Contusion Spinal Cord Injury Mouse Model
Source: Biomed Res Int. 2016 Sep 4;2016:9249401. doi: 10.1155/2016/9249401 (PMC5027055; doi:10.1155/2016/9249401)

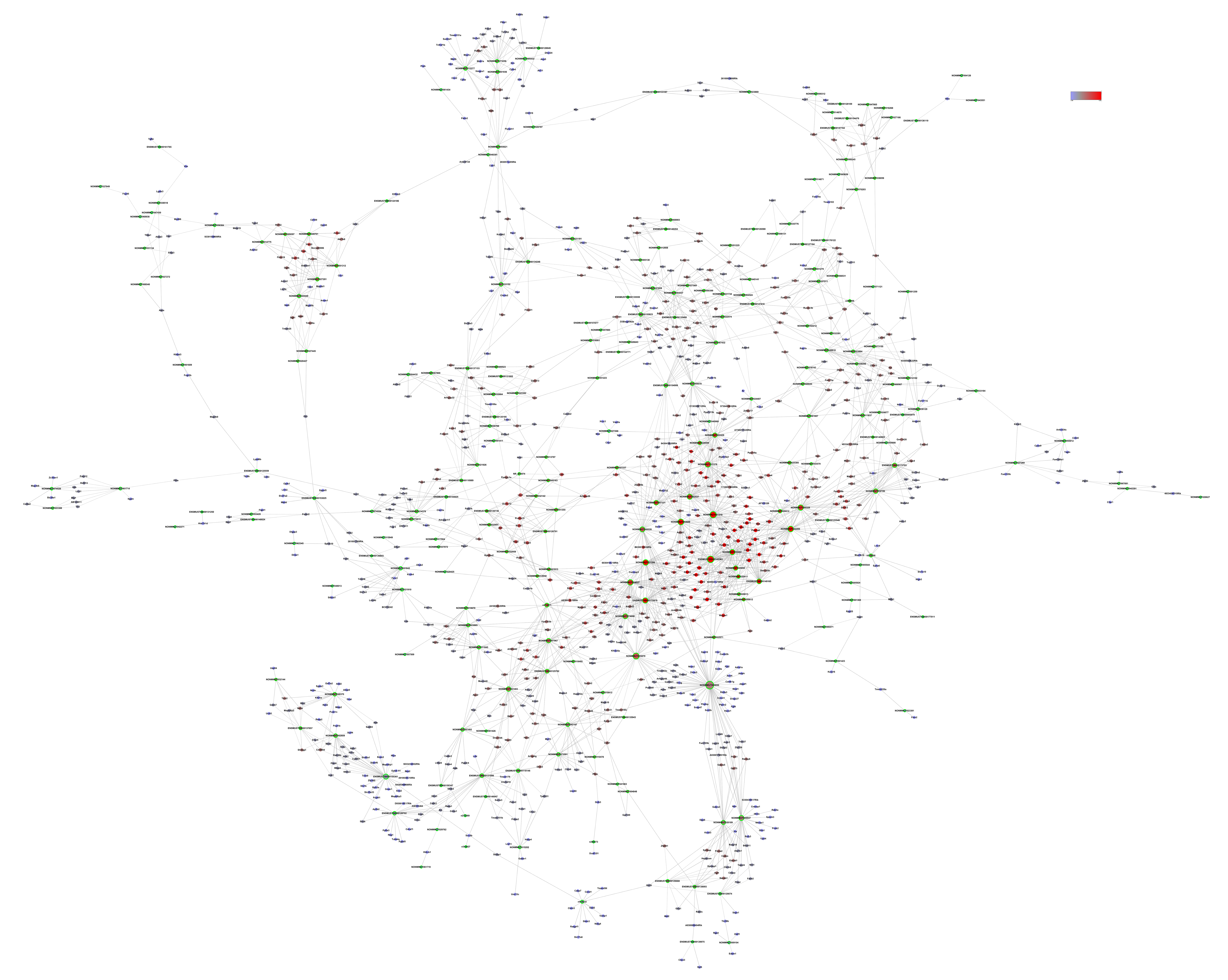

Supplement: Supplementary file 1 — S1 license pdf: The License Awarded by Soochow University. Table S1: Differential Expression of LncRNAs and Annotation. Table S2: Differential Expression of mRNAs and Annotation. Table S3: mRNAs STC Increase or Decrease. Table S4: LncRNAs STC Increase or Decrease. Table S5: The Significant GO Terms for Differentially Expressed. Table S6: The Significant Pathways of Differentially Expressed Genes. Table S7: The Quantity of the mRNA and LncRNA Network. S1 Fig: The dynamic network between LncRNAs and mRNAs. In this map, the circles with a green line represented LncRNAs, and the other circles represented mRNA. The lines between circles indicated regulation between these genes. The size of the circles indicated the ability of interaction between the genes. This ability was quantified by the degree, which was defined as the connective number between genes. The color was in accordance with the results of the clustering analysis, which was defined as the K-core. The same K-core indicated the similarity and correlation of function between genes. The red color representing the maximal K-core indicated the strongest regulation level in this network. [file 9249401.f1.zip › S1 figure.tif]
